# A test of stress, cues, and re-exposure to large wins as potential reinstaters of suboptimal decision making in rats

**DOI:** 10.3389/fpsyg.2015.00394

**Published:** 2015-04-07

**Authors:** Nina P. Connolly, Jung S. Kim, Brendan J. Tunstall, David N. Kearns

**Affiliations:** Department of Psychology, American University, Washington, DC, USA

**Keywords:** decision making, suboptimal choice, reinstatement, gambling, animal model, rats

## Abstract

The present experiment investigated potential reinstaters of suboptimal economic decision making in rats. Rats were first trained on a version of the rat Gambling Task under conditions designed to promote choice of a suboptimal option that occasionally resulted in large “wins” (four sucrose pellets). In a second phase, preference for this economically suboptimal option was reduced by substantially increasing the probability of punishment when this option was chosen. Then, three events were tested for their ability to reinstate choice of the suboptimal option. A brief period of re-exposure to a high frequency of large wins significantly increased choice of the suboptimal option. The pharmacological stressor yohimbine did not reinstate suboptimal choice, but did increase impulsive action as indexed by premature responding. Presentation of cues previously associated with large wins did not alter behavior. Results suggest reinstaters of suboptimal choice may differ from reinstaters of extinguished drug- and food-seeking behavior.

## Introduction

Recently, there has been much interest in studying decision making under conditions of uncertainty in animals (de Visser et al., 2011; Paglieri et al., 2014; Cocker and Winstanley, 2015; Zentall, 2015). A number of new tasks for rats have been developed to study choice between options that vary in terms of economic costs and benefits. These include the rat Gambling Task (rGT; van den Bos et al., 2006, 2012, 2013, 2014; Rivalan et al., 2009, 2013; Zeeb et al., 2009; Zeeb and Winstanley, 2011, 2013; Koot et al., 2012, 2013, 2014) and the probabilistic delivery task (PDT; Adriani and Laviola, 2006; Adriani et al., 2006, 2009, 2010; Koot et al., 2012; Zoratto et al., 2013, 2014). On these tasks, contingencies are arranged so that choice of the large reward option(s) is economically suboptimal. Because suboptimal decision making contributes to disordered gambling (Goudriaan et al., 2005; Roca et al., 2008; van Holst et al., 2010), use of these tasks in rats may model the factors that cause people to continue to gamble despite negative consequences (Potenza, 2009; Winstanley, 2011; Paglieri et al., 2014).

Previous studies with rats have shown that choice of a low probability or economically suboptimal large reward can be reduced by decreasing the probability of that reward to low (near 0) levels (Adriani et al., 2009, 2010; Zeeb et al., 2009; Kearns and Gomez-Serrano, 2011). The goal of the present study was to investigate the reinstatement of preference for a suboptimal large reward option after such preference had been eliminated in this manner. To the extent that rats’ maladaptive preference for economically suboptimal large reward models gambling-like behavior, identifying reinstaters of such preference could provide insight into factors that produce relapse to disordered gambling.

The present study used a version of the rGT where rats made nose-poke responses to choose among four options associated with different risk/reward payoffs. When rats “won,” they received sucrose pellets. When they “lost,” they experienced a punishment timeout period that prevented them from earning more sucrose pellets for a fixed period of time. Each option was associated with a different probability of winning, number of sucrose pellets, and length of punishment period. In the present experiment, rats were first trained under conditions designed to promote choice of an economically suboptimal large reward associated with long timeout periods instead of smaller, more frequent reward. Then, rats’ choice of this suboptimal option was reduced (via a greatly decreased probability of winning) prior to testing for reinstatement.

Three events—stress, cues, and re-exposure to large wins—were tested for their ability to reinstate choice of the suboptimal option. The putative reinstaters were designed to be similar to three events known to produce a reappearance of behavior after extinction in the reinstatement paradigm (Shaham et al., 2003). The Stress group received an injection of the pharmacological stressor yohimbine, which has been shown to reinstate extinguished drug- and food-seeking behavior (e.g., Feltenstein and See, 2006; Nair et al., 2006, 2008). The Cues group received presentations of a tone stimulus that was previously paired with large wins. Reward-associated cues have been shown to be reliable reinstaters of food- and drug-seeking behavior (e.g., Buffalari et al., 2013; Tunstall and Kearns, 2014). The Re-exposure group experienced a brief series of consecutive large wins at the start of the test session. Brief, response-contingent re-exposure to the event that previously maintained behavior has been shown to be an effective reinstater of alcohol- and cocaine-seeking behavior (Chiamulera et al., 1995; Kruzich, 2007). The primary question of the present experiment was whether these three kinds of events would lead to a reinstatement of choice of the economically suboptimal large reward option.

## Materials and Methods

### Subjects

Thirty-six naïve adult male Long-Evans rats completed the experiment. Three other rats began the experiment but did not complete it. Two of these were dropped because they could not acquire the nose-poke response in a timely manner and one was dropped because it displayed a persistent avoidance of Hole D throughout training. Rats were individually housed in plastic cages with wood-chip bedding and metal wire tops. They were maintained at 85% of their free-feeding weights (approximately 350–450 g) throughout the experiment by feeding rats after each session an amount of rat chow that accounted for the number of sucrose pellets they earned during the session. Rats had unlimited access to water in their home cages. The colony room where the rats were housed had a 12-h light:dark cycle with lights on at 08:00 h. Training sessions were conducted 5–7 days per week during the light phase of the light:dark cycle. Throughout the experiment, rats were treated in accordance with the Guide for the Care and Use of Laboratory Animals (National Academy of Sciences, 2011) and all procedures were approved by American University’s Institutional Animal Care and Use Committee (IACUC).

### Apparatus

Training took place in five Med-Associates (St. Albans, VT, USA) modular test cages (30.5 cm × 24.1 cm × 29.2 cm). The side walls were made of clear polycarbonate and the front and rear walls were aluminum. Each chamber had a grid floor. There were five nose-poke holes located on the back wall of the chamber. Each nose-poke hole was 2.5 cm × 2.5 cm square. Nose-poke holes were spaced approximately 2.0 cm from each other and 1.5 cm from the floor. A photobeam spanned the hole horizontally and would record a nose-poke if the rat inserted its nose 1.0 cm into the hole. A recessed LED cue-light was located at the back of each nose-poke hole. The center nose-poke hole was not used in the present experiment. A food trough was located in the center of the front wall of the chamber. A photobeam would record an entry if a rat put its nose 1.0 cm into the trough. The food trough could be illuminated by a 100-mA lightbulb located within it. A 100-mA houselight was located on the front wall and near the ceiling of the chamber. A Sonalert tone generator, located in the center of the ceiling of the chamber, was used to provide a tone stimulus (4500 Hz, 85 dB). Each operant chamber was located within an attenuation chest equipped with a ventilation fan that circulated the air and provided masking noise.

### Procedure

#### Phase 1: Promoting Hole D Choice on the Gambling Task

***Acquisition***

Due to a limited number of training chambers, the experiment was run in two replications, with half the subjects of each group (or as close to half as possible in groups with odd numbers of subjects) run in each of the two replications. Rats were first trained to acquire the nose-poke response. For the first half of rats trained, initially a discrete-trials acquisition procedure was used where, on each trial, a randomly selected nose-poke hole was illuminated for 10 s. A nose-poke in the lighted hole resulted in delivery of a single sucrose pellet (Bioserv, Flemington, NJ, USA, Product #F07257, 45-mg Banana Flavored Sucrose Pellets, 0.18 kcal/pellet). Failure to make a nose-poke was recorded as an omission and initiated a 5-s intertrial interval (ITI) where no hole was illuminated. Sessions lasted for 30 min or until rats obtained 100 sucrose pellets. The goal was to train rats on this procedure until they met a criterion of obtaining at least 90 sucrose pellets within a session. The intention of this 10-s discrete trials acquisition procedure was make rats learn that the cue-light signaled the availability of reward. On the final version of the task, trials were signaled by the illumination of the cue-lights. We wanted rats to be able to complete many trials over the course of the session, so we thought that initially training them to quickly make a response soon after cue-light illumination would help in this regard.

Because many rats had difficulty acquiring the nose-poke response under these conditions, the length of nose-poke hole illumination was extended from 10 s to up to 120 s. Most rats ultimately required sessions where a nose-poke hole remained illuminated for the entire session and nose-pokes in it were reinforced on a fixed-ratio (FR) one schedule until the subject obtained 100 pellets or 60 min elapsed. Once they learned to nose-poke, rats receiving these remedial acquisition procedures were returned to the 10-s discrete-trials program and trained on it until they reached the criterion of obtaining at least 90 sucrose pellets within a 30-min session.

Because it was apparent after running the first half of rats that using the 10-s discrete trials procedure from the outset was inefficient for nose-poke acquisition, the second half of rats were trained on a modified procedure for the first four sessions of training. For these rats, nose-poke acquisition began with the cue-light in the left-most hole on continuously for the duration of a session. Rats could nose-poke in that hole for sucrose pellets on an FR-1 schedule. Sessions lasted for 1 h or until subjects obtained 100 pellets. On the next session, rats were then trained on the same procedure but with the next nose-poke hole to the right illuminated. Training on this procedure continued until rats received one session with each of the four nose-poke holes. As all rats had acquired the nose-poke response by the fourth session, they were then placed on the 10-s discrete trials procedure described above and trained on that procedure until meeting the same acquisition criterion described for the first half of rats trained. In future experiments, we will avoid use of the 10-s discrete trials acquisition procedure and start with the modified acquisition procedure described above where rats could make a reinforced nose-poke response at any time.

***Forced-choice***

Rats were then trained for seven sessions on a forced-choice procedure where they learned the different outcomes associated with the different holes on the gambling task. For half the rats, the four holes from left to right (from the rats’ perspective while facing the holes) were designated A, B, C, and D; for the other half, they were designated D, C, B, and A. Table 1 presents the contingencies associated with each hole. A randomly selected hole was illuminated and if the rat made a nose-poke response in that hole only within 10 s, the outcome associated with that hole occurred. If it was a reward trial (a “win”), the sucrose pellet(s) were delivered and then a 5-s ITI began. Additionally, delivery of the large, 4-pellet reward associated with Hole D wins was accompanied by a 5-s tone stimulus. The 5-s tone began as soon as the rat made the nose-poke response in Hole D and continued during pellet delivery. This “big win” cue was used in later tests of cue-induced reinstatement. If a trial was a punishment trial (a “loss”), a response did not result in pellet delivery, but, instead, the cue-light inside the hole flashed (0.5 s on/0.5 s off) for a 5-, 10-, 30-, or 40-s punishment timeout period that delayed the initiation of the next trial and thereby reduced the total number of sucrose pellets that could be earned. At the end of the punishment period, the light inside the food trough was illuminated but no pellets were delivered. A head-entry response in the food trough was required to turn off the trough light and initiate the next trial, which was preceded by a 5-s ITI. The light inside the food trough was also illuminated on win trials. On both win trials and loss trials, it remained illuminated until the rat made a head-entry response in the food trough. This response initiated the 5-s ITI period that preceded the next trial. This contingency was programmed on both trial types so that the rat was always in the same place (i.e., at the food trough) at the start of the 5-s ITI preceding the next trial. Nose-pokes in holes other than the illuminated one during a trial were recorded as incorrect responses, but had no scheduled consequences. Failures to make a response during the 10-s trial were recorded as omissions. Premature nose-poke responses during the ITI before a hole was illuminated were recorded, but had no scheduled consequences during this phase.

**TABLE 1:** **Gambling task contingencies associated with each hole during Phase 1**.

Nose-poke hole	A	B	C	D	D*	D**
Number of pellets	1	2	3	4	4	4
Probability of punishment (timeout)	0.1	0.2	0.5	0.4	0.2	0.9
Punishment timeout duration (seconds)	5	10	30	40	40	40
Number of pellets per 30-min session	295	411	135	206	443	18

Choice of Hole D was the target behavior in the present study. In previously used versions of the gambling task, the probability of a loss on Hole D trials is 0.6 (e.g., Zeeb and Winstanley, 2011). With the loss probability set to 0.6, rats choose Hole D on only about 10–15% of trials (Zeeb and Winstanley, 2011). It is difficult to study the reinstatement of a behavior that occurs at such a low baseline rate. To best approximate the reinstatement paradigm, the goals of the present procedures were to induce moderate to high preference for Hole D in Phase 1, and then reduce Hole D preference to low levels in Phase 2 prior to reinstatement testing. Thus, to boost rates of Hole D choice in Phase 1, the loss probability was reduced from 0.6 to 0.4. Despite this change, Hole D remained a disadvantageous option because a maximum of only 206 pellets could be earned by consistently choosing it (see Table 1). This is only half the number of pellets that could be earned by consistently choosing Hole B, the optimal choice, and only about two-thirds the number of pellets that could be earned by consistently choosing Hole A. Furthermore, the risk of losing on any given trial was still at least twice that of either Hole A or Hole B and losses on Hole D trials resulted in the longest punishment timeout periods (40 s) among all the options. The choice of whether to study the reinstatement of Hole C (the other suboptimal option) or Hole D was arbitrary. Previous studies (e.g., Zeeb et al., 2009) found that Hole C is chosen on only approximately 10% of trials when its loss probability is set to 10%. It would not be possible to study the reinstatement of a behavior that occurs at such a low baseline rate. Thus, it would have been necessary to change the loss probability associated with Hole C in the same way that the loss probability of Hole D was adjusted to promote choice of it in Phase 1, before eliminating it in Phase 2 prior to reinstatement testing.

***Free-choice***

Following the seven forced-choice sessions, free-choice training began. Now, all four nose-poke holes were illuminated on each trial and the rat was free to choose which hole to respond in. The outcomes associated with each hole remained as presented in Table 1. During free-choice training, premature responses (i.e., those made during the ITI prior to the start of a trial) resulted in a 5-s timeout period signaled by illumination of the houselight. When the houselight was turned off, the light inside the food trough was illuminated. A head entry response was required to turn off this light and initiate the next trial, which was preceded by a new 5-s ITI. Rats were trained on the free-choice procedure for a minimum of 12 sessions and until at least 50% of the choices were for Hole D for two consecutive sessions. If a rat displayed a low preference for Hole D, it was trained on a modified forced-choice procedure where the probability of punishment on Hole D was reduced to 0.2 for three sessions. The column labeled D* in Table 1 shows the expected number of pellets that could be earned by consistently choosing Hole D with this reduced loss probability. Approximately half of the subjects in each group received this brief, three-session forced-choice intervention intended to boost Hole D choice during the free-choice portion of Phase 1. The exact numbers were 4/10, 5/10, 3/7, and 4/9 for the Control, Stress, Cues, and Re-exposure groups, respectively. These rats were then returned to the regular free-choice procedure for a minimum of six sessions and until meeting the 50% Hole D choice criterion, or until having a maximum of 20 free-choice sessions.

#### Phase 2: Reducing Hole D Choice

The goal of Phase 2 was to reduce choice of Hole D to low levels by increasing the probability of punishment to 0.9. This probability was chosen because the goal was to reduce Hole D choice to low levels, but not completely eliminate it. All other aspects of the procedure were the same as described in Section “Free-choice.” The column labeled D** in Table 1 shows the expected number of pellets that could be earned by consistently choosing Hole D with the loss probability set to 0.9. Rats were trained on the Phase 2 procedure for a minimum of six sessions and until choice of Hole D was less than 15% for two consecutive sessions.

#### Reinstatement Testing

Once meeting the Phase 2 criterion, rats were assigned to one of four groups: Stress (*n* = 10), Cues (*n* = 7), Re-exposure (*n* = 9), or Control (*n* = 10). Group assignment was made with the goal of matching groups as much as possible in terms of percent choice of Hole D on the final sessions from Phases 1 and 2 after first counterbalancing numbers of rats trained with the holes designated A, B, C, and D from left to right vs. right to left. For all groups, the procedural variables of the gambling task during the reinstatement test session were the same as in Phase 2. Only events preceding this session differed over groups.

Rats in the Stress group were given an i.p. injection of 2.0 mg/kg yohimbine (Akorn, Inc., Decatur, IL, USA). The yohimbine was dissolved in sterile saline at a concentration of 2.0 mg/ml. Following the yohimbine injection, rats waited in their homecages for a 30–45 min period prior to the start of the test session. Previous yohimbine-induced reinstatement studies (e.g., Nair et al., 2006) have used this delay period.

Rats in the Cues group were placed into the rGT chamber and were exposed to 16 presentations of the tone stimulus previously associated with wins on Hole D. Each tone presentation lasted for 5 s. Tone presentations were separated by 25-s periods where the tone was off. The rGT session began 25 s after the final tone presentation.

Rats in the Re-exposure group were placed into the chamber and received 10 Hole D forced-choice trials where every trial was a win. Each trial started with illumination of the cue-light inside Hole D. The light remained on until the rat made a response. Trials were separated by ITIs lasting 5 s. The gambling task began once the rat broke the photobeam in the food trough upon collecting the sucrose pellets delivered on the final Hole D forced-choice trial.

Rats in the Control group were treated the same as rats in the Yohimbine group, except they received an i.p. injection of saline rather than yohimbine. The volume of the saline injection was equivalent to the volume of injections received by rats in the Yohimbine group.

### Data Analysis

The primary measure of interest was the change in percent of Hole D choices on the reinstatement test as compared to the final Phase 2 session. The percentage of choices made on the other holes, total numbers of choices completed, numbers of premature responses, and numbers of omissions were also analyzed. In addition to whole-session level data, results from the first 10 choices of the reinstatement test were examined separately to determine whether there were any short-lived effects of the putative reinstaters. For all statistical tests, α = 0.05. One-way, repeated measures, or mixed ANOVAs, followed by paired-samples *t*-tests, where appropriate, were performed. Because percent choices of the holes are non-independent measures, separate one-way or repeated measures ANOVAs were performed on the percentage measures for each hole.

## Results

### Phase 1

Rats required approximately eight sessions to acquire the nose-poke response (group means ranged from 7.4 to 8.8 and did not significantly differ, *F* < 1). This was followed by seven forced-choice sessions for all rats. Rats then had an average of approximately 13 sessions on the free-choice gambling task procedure (group means ranged from 12.4 to 14.6, no difference among groups, *F* < 1).

**FIGURE 1:**
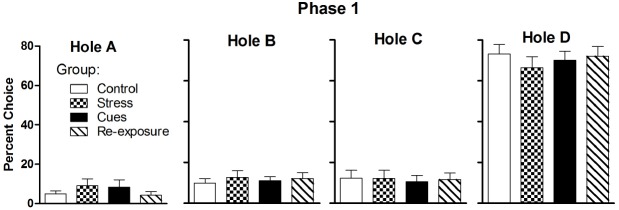
**Mean (± SEM) percent choices of each hole made by each group averaged over the final two sessions of Phase 1.** The probability of a “loss” (punishment) on Hole D was 0.4 during Phase 1.

Figure 1 presents percent choice for each hole averaged over the final two sessions in Phase 1. The strategy to promote Hole D preference by using a 0.4 probability of a loss was successful, as rats chose it on approximately 70% of trials. Rats chose the other holes on only approximately 5–15% of the time, on average. There were no differences among groups for any of the holes (all *F*s < 1). Group mean total numbers of choices made on these sessions ranged from 60.4 to 67.1 (no group differences, *F* < 1) and the mean numbers of sucrose pellets earned ranged from 128.5 to 143.8 (no group differences, *F* < 1). The group mean numbers of omissions ranged from 7.1 to 10.5 (no group differences, *F* < 1).

#### Phase 2

**FIGURE 2:**
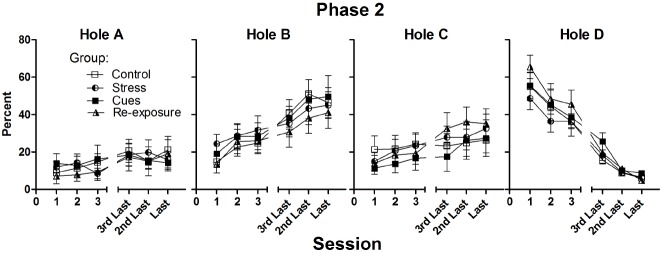
**Mean (± SEM) percent choices of each hole made by each group on the first three and on the last three sessions of Phase 2.** The probability of a loss on Hole D was 0.9 throughout Phase 2.

Figure 2 presents percent choices of each hole during the first three sessions and last three sessions of Phase 2 (numbers of Phase 2 sessions varied over rats), where the probability of punishment on Hole D was increased to 0.9. Rats required approximately nine sessions on the Phase 2 procedure to meet the criterion of choosing Hole D on less than 15% of trials on two consecutive sessions (group mean number of sessions ranged from 7.9 to 9.9, no difference among groups, *F* < 1).

Choice of Hole D fell from approximately 70% on the final Phase 1 session to a mean of less than 10% for all groups on the final Phase 2 session. Choice of the other holes, especially Hole B, increased over these sessions. These impressions were confirmed by 4 × 6 (Group × Session) repeated measures ANOVAs. For each hole, there was a significant main effect of Session [all *F*(5,160)s ≥ 4.6, all *p*s ≤ 0.001], but no main effect of Group (all *F*s < 1) or Group × Session interaction (all *F*s < 1).

Because by the end of Phase 2 rats chose Hole D rarely, they only infrequently experienced the relatively long punishment timeout periods associated with losses on that hole. This allowed rats to make more total choices per session than they did in Phase 1. On the final Phase 2 session, rats’ mean total numbers of choices made per session ranged from 85.6 to 96.5, depending on group (no group differences, *F* < 1). This compares with only approximately 60–65 choices per session during Phase 1, when rats chose Hole D more frequently. The group mean numbers of sucrose pellets earned on the final Phase 2 session ranged from 117.4 to 132.0 (no group differences, *F* < 1). The group mean numbers of omissions on the last Phase 2 session ranged from 3.8 to 11.0 and did not differ by group [*F*(3,32) = 1.0, *p* = 0.40].

#### Reinstatement Test

**FIGURE 3:**
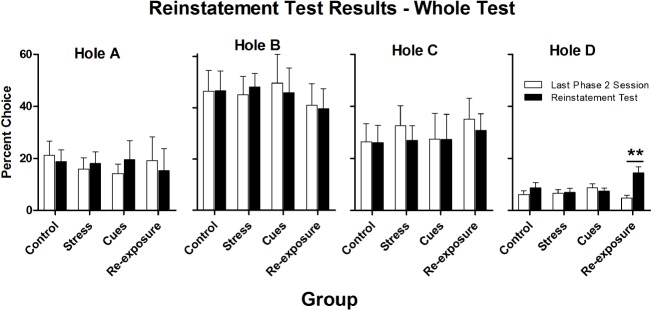
**Mean (± SEM) percent choices of each hole made by each group on the final Phase 2 session (white bars) and on the reinstatement test (black bars).** **p < 0.01.

**FIGURE 4:**
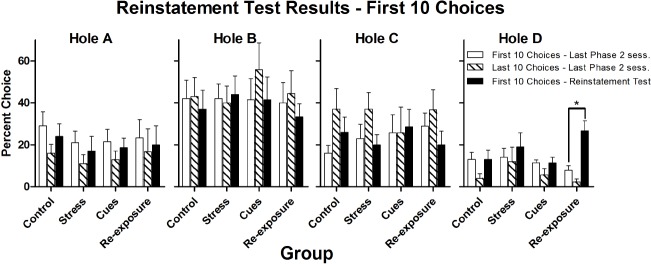
**Mean (± SEM) percent choices of each hole made by each group on the first 10 choices of the final Phase 2 session (white bars) and on the first 10 choices of the reinstatement test (black bars).** *p < 0.05.

Figure 3 presents percent choices of each hole on the reinstatement test and on the last day of Phase 2. Choice of Hole D was the behavior of interest. The only group for which there was an increase in Hole D choice over sessions was the Re-exposure group. For all other groups, there was no change in choice behavior from the last Phase 2 session to the reinstatement test. The first 10 choices of these two sessions were examined separately to see if any of the putative reinstaters produced a short-lasting increase in Hole D choice. Figure 4 presents these data, which generally parallel those from the whole test.

Statistical analyses confirmed the impressions described above. Separate 4 × 2 (Group × Session) repeated measures ANOVAs performed on the whole session data and on the first-10 choices data from the final Phase 2 session and the reinstatement test indicated that only for Hole D was there a significant Group × Session interaction [whole test, *F*(3,32) = 6.0, *p* = 0.002; first 10 choices, *F*(3,32) = 3.6, *p* = 0.03]. There was also a main effect of Session for Hole D [whole test, *F*(1,32) = 8.6, *p* < 0.01; first 10 choices, *F*(1,32) = 6.3, *p* = 0.02]. Subsequent paired-samples *t*-tests confirmed that only the Re-exposure group significantly increased choice of Hole D over sessions [whole test, *t*(8) = 3.6, *p* < 0.01; first 10 choices, *t*(8) = 3.7, *p* < 0.01; for all other groups, all *t*s ≤ 1.0, *p*s ≥ 0.21]. For no other hole was there a significant Group × Session interaction [*F*(3,32)s ≤ 2.0, *p*s ≥ 0.14] or a main effect of Session [*F*(1,32)s < 2.3, *p*s ≥ 0.14]. For no hole was there a main effect of Group (all *F*s < 1).

When the last 10 choices of the final Phase 2 session were compared to the first 10 choices of the test, there were significant main effects of Session for Hole C and Hole D [*F*(1,32)s ≥ 4.6, *p*s < 0.05], with Hole C responding being generally higher on the last 10 choices of the final Phase 2 session than during the first 10 choices of the test and Hole D responding being generally lower on the final 10 choices of the last Phase 2 session as compared to the first 10 choices of the test. There was no main effect of session for Holes A and B [*F*(1,32)s ≤ 4.1, *p*s > 0.05]. There were no significant Group × Session interactions [*F*(3,32)s ≤ 1.5, *p*s > 0.23] or main effects of Group [*F*(3,32)s ≤ 1.4, *p*s > 0.25] for any hole.

**FIGURE 5:**
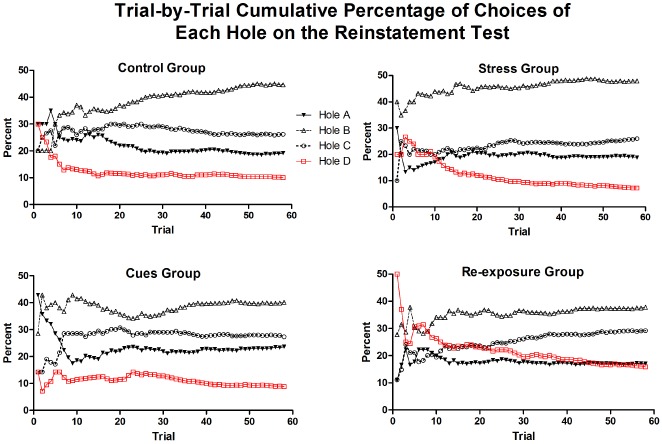
**Mean cumulative percentage of choices of each hole over the first 58 choices of the reinstatement test for each group.** Fifty-eight choices are shown because that is the minimum number of choices completed on the test by all rats.

To provide some information on the duration of reinstatement effects, Figure 5 presents for each group the cumulative percentages of choices made in each hole over each of the first 58 choices of the test (58 was the minimum number of choices made on the test by all rats). This figure shows that the Control, Stress, and Cues groups were highly similar in their distributions of choices over holes. (The apparently large variability over the first few choices is due to the small numbers of choices on which the percentage is based early in the test.) As can be seen in the results of the Re-exposure group, the effect of the re-exposure treatment was relatively brief. The Re-exposure group maintained heightened choice of Hole D (relative to the other groups) through approximately trial 20. After that point, the distribution of responses over holes began to approximate the distributions observed in the other groups.

**FIGURE 6:**
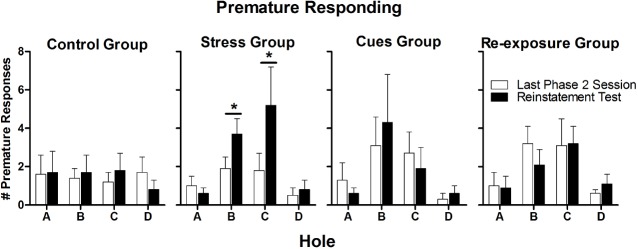
**Mean (± SEM) numbers of premature responses made by each group on the final Phase 2 session (white bars) and on the reinstatement test (black bars).** *p ≤ 0.05.

Figure 6 presents numbers of premature responses for each hole on the reinstatement test and on the final Phase 2 session. Yohimbine administration in the Stress group approximately doubled the numbers of premature responses on Holes B and C. There was not a similar increase in premature responding in any of the other groups. A 4 × 4 × 2 (Group × Hole × Session) mixed ANOVA performed on the numbers of premature responses indicated that there was a significant Group × Hole × Session interaction [*F*(9,96) = 2.4, *p* = 0.02]. To further resolve this interaction, separate 4 × 2 (Hole × Session) repeated measures ANOVAs were performed for each group. For the Stress group only, there were significant main effects of Session [*F*(1,9) = 5.5, *p* = 0.04] and Hole [*F*(3, 27) = 4.4, *p* = 0.01] as well as a significant Session × Hole interaction [*F*(3,27) = 4.1, *p* = 0.02]. Subsequent paired-samples *t*-tests indicated that there was a significant increase in premature responding over sessions for Hole B [*t*(9) = 3.0, *p* = 0.01] and a marginally significant increase for Hole C [*t*(9) = 2.3, *p* = 0.05]. For none of the other groups were there significant effects of Session (all *F*s < 1), Hole (all *F*s ≤ 2.7, *p*s ≥ 0.07), or their interaction (all *F*s ≤ 1.5, *p*s ≥ 0.25).

The mean number of total choices made on the reinstatement test ranged from 85.3 to 93.3, depending on group. There were no differences among groups (*F* < 1). The mean number of omissions ranged from 4.0 to 11.6 and also did not differ among groups (*F* < 1).

## Discussion

The present experiment sought to determine whether the same kinds of events known to reinstate extinguished food- and drug- seeking behavior would also reinstate economically suboptimal decision making. After choice of a suboptimal large reward option was reduced to low levels, there was evidence that re-exposure to several large “wins” temporarily increased choice of that option. There was no evidence that the pharmacological stressor yohimbine or that cues previously associated with large wins reinstated suboptimal choice. Thus, the present study found that only one of the three types of events that reinstate extinguished food- and drug-seeking behavior in rats would also reinstate suboptimal decision making.

One potential concern about the lack of an effect of yohimbine on choice here is that the dose was too low or too high. There are two reasons that this possibility seems unlikely. First, yohimbine significantly increased premature responding, indicating that the dose used here was large enough to be behaviorally active, but was not so large that it suppressed behavior. Second, the dose used here—2.0 mg/kg i.p.—has been shown in previous studies to be a highly effective reinstater of food seeking (Ghitza et al., 2006; Nair et al., 2006, 2008). In fact, 2.0 mg/kg was found to be the most effective dose among the range of doses tested by Nair et al. (2006). Other studies investigating the reinstatement of drug seeking have found doses from 1.25 to 2.5 mg/kg to be effective reinstaters (e.g., Shepard et al., 2004; Feltenstein and See, 2006; Banna et al., 2010).

The increase in premature responding produced by yohimbine replicates results of previous studies showing that yohimbine increases premature responding on the 5-choice serial-reaction-time task (5-CSRTT; Sun et al., 2010; Torregrossa et al., 2012). Premature responding on the 5-CSRTT has been considered a measure of impulsive action (Robbins, 2002; Winstanley et al., 2004). On both the 5-CSRTT and the rGT, the rat is required to wait until a cue-light inside a nose-poke hole is illuminated before making a nose-poke response. On both tasks, nose-poking before the cue-light illumination results in a timeout that delays the next trial. Because of these procedural equivalences, premature responding on the rGT can also be considered a measure of impulsive action. The results of the Stress group, where yohimbine increased premature responding but did not alter hole choice, suggest that changes in impulsive action do not necessarily translate into changes in risk/reward preferences. This outcome is consistent with previous studies finding that impulsive action is unrelated to choice between different reward options (Winstanley et al., 2004; Lovic et al., 2011; Baarendse and Vanderschuren, 2012; Broos et al., 2012).

A limitation of this study is that only one kind of stressor—yohimbine—was used. Alternative methods of producing a stress response in rats include exposure to footshock, restraint stress, forced swimming, or administration of corticosterone. There is evidence that different stressors may affect behavior differently (Armario et al., 1991; Noori et al., 2014). Yohimbine was chosen here because it has been well established that it reliably produces reinstatement of previously extinguished behavior (Feltenstein and See, 2006; Nair et al., 2006, 2008). Future research will be necessary to determine if different results might have been obtained in the present study if different stressors had been used. A second possible limitation of the current study is that the rats were trained and tested during the light phase of the light/dark cycle. It is possible that different results would be obtained if the experiment were conducted during the dark phase of the cycle. Some studies have found that stress may produce different effects in rats during the dark phase as compared to during the light phase (e.g., Verma et al., 2010). Perhaps the Stress group would have behaved differently on the test if it were conducted during the dark phase. Future research will be needed to answer this question. Another possible limitation concerns the procedure used for the Control group. For rats in this group (and for those in the Stress group), the test session began shortly after placement in the chamber. In contrast, for rats in the Cues group, there was an 8-min pre-test period that rats spent in the chamber when they experienced cues. The Re-exposure group also had a brief period of exposure to the chamber prior to the start of the test as they completed their 10 Hole D win trials. A control group that received 8 min of exposure to the chamber alone prior to the test would have more closely matched the experiences of the rats in the Cues and Re-exposure groups. However, evidence suggesting that a few min of exposure to the chamber prior to the test would not have altered behavior comes from the test results of the Cues group, which had 8 min of exposure to the chamber (plus intermittent tone presentations) but whose behavior on the test was essentially the same as that of the Control group as well as that of the Cues group’s own final Phase 2 session (which did not begin with 8 min exposure to the chamber).

Presentation of a tone cue paired with Hole D large wins had no effect on behavior during the reinstatement test. This was somewhat surprising because in the drug reinstatement paradigm, which the design of the present experiment was based on, cues are reliable reinstaters of food or drug seeking in rats (e.g., Fuchs et al., 2004a,b; Tunstall and Kearns, 2014). A possible reason for the lack of an effect of the cue is that the tone frequency or intensity was too high or too low to be perceived by the rats. But previous studies from our lab have shown that the same tone used in the present experiment functioned as an effective trigger for the recovery of extinguished cocaine seeking in rats (Kearns et al., 2012; Tunstall et al., 2013). Thus, it seems unlikely that the physical aspects of the cue prevented it from affecting behavior. It is possible, however, that a cue with a different functional role could have served as a reinstater. In the present study, the tone was presented simultaneously with the delivery of the four sucrose pellets received on Hole D wins. This made the tone functionally comparable to the injection-paired conditioned stimulus (CS) used in drug cue-induced reinstatement studies. Cues can also serve as discriminative stimuli (S^D^s) or as contextual cues. Previous studies in rats have shown that while CSs, S^D^s, and contextual cues can all potentially reinstate drug seeking (e.g., Alleweireldt et al., 2001; Ciccocioppo et al., 2004; Fuchs et al., 2004a; Kearns and Weiss, 2007, 2012), there are important differences among these types of cues in terms of underlying neural mechanisms and effects on behavior (e.g., Di Ciano and Everitt, 2003; Bossert et al., 2007; Chaudhri et al., 2009). Future research is necessary to determine whether different functional types of cues (e.g., S^D^s or contextual cues) might reinstate suboptimal decision making.

### Conflict of Interest Statement

The authors declare that the research was conducted in the absence of any commercial or financial relationships that could be construed as a potential conflict of interest.
